# The current landscape, advancements, and prospects in the treatment of patients with EGFR exon 20 insertion mutations warrant scientific elucidation

**DOI:** 10.3389/fonc.2024.1367204

**Published:** 2024-06-11

**Authors:** Xiuyue Man, Xueru Sun, Chen Chen, Yan Xiang, Jing Zhang, Lei Yang

**Affiliations:** Cancer Center, The First Hospital of Jilin University, Changchun, Jilin, China

**Keywords:** lung cancer, EGFR exon 20 insertion mutations, treatment, chemotherapy, immunotherapy, targeted therapy

## Abstract

Epidermal growth factor receptor (EGFR) exon 20 insertion (ex20ins) mutations are the third most prevalent mutation in non-small cell lung cancer (NSCLC), following the 19del and L858R mutations. The unique nature of the EGFR ex20ins mutation poses challenges for the effectiveness of first- and second-generation EGFR tyrosine kinase inhibitors (TKIs). As a result, chemotherapy remains the primary and more effective treatment approach. However, with advancements in time and technology, numerous experimental studies have revealed the potential of novel drugs and therapies to have stronger inhibitory effects on EGFR ex20ins mutations. In this comprehensive review, we provide an overview of the current treatment landscape, recent advancements, and the prospects for patients with advanced NSCLC characterized by EGFR ex20ins mutations.

## 1 Introduction

Lung cancer is a highly lethal malignancy, with non-small cell lung cancer (NSCLC) comprising about 75% to 80% of all lung cancer cases (1–3). Usually, the disease remains asymptomatic in its early stages, often leading to late-stage diagnosis, which is less amenable to surgical intervention. However, the advent of targeted therapies has significantly improved survival rates for NSCLC patients. The most commonly observed mutated genes in lung cancer include p53, KRAS, EGFR, MET, and ALK (4–6). Among these, the epidermal growth factor receptor (EGFR) is a pivotal oncogenic driver, particularly in the Asian population, where it is found in 30% to 50% of cases, compared to 10% to 20% in Caucasian populations (7, 8). Classic EGFR mutations include exon 19 deletions (19del, constituting approximately 45% of EGFR mutations) and exon 21 point mutations (L858R, accounting for about 40% of EGFR mutations) (9, 10), collectively representing 80% to 90% of EGFR gene mutations. Additionally, the third most prevalent EGFR mutation is the exon 20 insertion mutation, known as EGFR ex20ins (11–13), which predominantly occurs in female, non-smoking individuals, and in adenocarcinoma tissue (13, 14). This mutation typically resides within the N-terminal region of the EGFR tyrosine kinase domain after the C-helix and can be categorized into C-helix insertions (3%-6%), proximal loop insertions (70%), and distal loop insertions (30%) (7, 15, 16). These insertions promote active kinase conformation and often display mutual exclusivity with other mutations. EGFR ex20ins represents a combination of in-frame insertions and/or duplications spanning 3 to 21 base pairs, mainly clustering between codons 767 and 774, with some also occurring between codons 762 and 764 (17).To date, over 60 different mutation variants have been identified (13), with the most prevalent insertion being V769_D770insASV, accounting for approximately 22% of all EGFR ex20ins mutations (18). Other common EGFR ex20ins mutations include D770_N771>ASVDN (21%), N771_P772>SVDNP (20%), and N771_H773dupNPH (8%), collectively representing around 50% of EGFR ex20ins mutations (19). The exact prevalence of EGFR ex20ins in NSCLC patients remains unclear, but based on previous research, it is estimated to account for approximately 4% to 12% of all EGFR mutations in this patient population (7, 16).

Patients with EGFR exon 20 insertion mutations in non-small cell lung cancer (NSCLC) exhibit a less favorable prognosis compared to those with common EGFR mutations, with a median overall survival (mOS) of 16.5 months versus 33.0 months (13). When contrasted with NSCLC patients possessing rare EGFR mutations, the mOS is 16.8 months versus 22.5 months (20). However, the mOS for EGFR exon 20 insertion patients is similar to that of NSCLC patients without EGFR mutations, approximately 20.0 months (13). The diminished sensitivity of EGFR exon 20 insertion mutations to first-generation or second-generation EGFR tyrosine kinase inhibitors (EGFR-TKIs) (20, 21) in contrast to EGFR 19del and EGFR L858R mutations, is attributed to structural alterations within the EGFR kinase domain (7, 16). This resistance-inducing mutation is colloquially referred to as a “resistance mutation,” excluding the A763–764insFQEA variant, which accounts for approximately 6% of EGFR exon 20 insertions. Findings from a multicenter trial (22) reveal that, in the first-line treatment with EGFR-TKIs, patients harboring EGFR exon 20 insertion mutations exhibit a median progression-free survival (mPFS) of 5.7 months and an mOS of 19.0 months. In contrast, non-EGFR exon 20 insertion patients display an mPFS of 9.0 months, and an mOS of 29.9 months.

Chemotherapy remains the standard of care for first-line or second-line treatment of EGFR ex20ins mutation, and it has demonstrated superior efficacy compared to EGFR TKIs and immunotherapy. A study conducted in South Korea (23) involving 27 advanced NSCLC patients with EGFR ex20ins insertions revealed a median overall survival (mOS) of 29.4 months. Among these patients, 22 individuals received platinum-based systemic chemotherapy (e.g., cisplatin or carboplatin), achieving an objective response rate (ORR) of 50.0%, a disease control rate (DCR) of 77.2%, a median progression-free survival (mPFS) of 4.2 months, and an mOS of 29.4 months. Six patients were treated with EGFR-TKIs. Among these, three received erlotinib, two received afatinib, and one received osimertinib. Four patients had EGFR exon 20 insertions as the sole mutation, with three demonstrating progressive disease (PD) and one showing stable disease (SD). The other two patients had double mutations, including exon 20 insertion and an additional EGFR mutation, leading to a partial response. In a study by Ji (24)(**Table 1**) and colleagues, which involved 109 EGFR ex20ins NSCLC patients, 39 patients received platinum-based chemotherapy, achieving an ORR of 43%, a mPFS of 6.9 months, and a mOS of 31.0 months. Twenty-three patients were treated with conventional EGFR TKIs, with an ORR of 13%, a mPFS of 3.4 months, and a mOS of 31.0 months. Additionally, 23 patients received single-agent immune checkpoint inhibitors, with an ORR of 4%, a mPFS of 2.6 months, and a mOS of 30.8 months. Patients who underwent chemotherapy exhibited significantly improved ORR, mPFS, and mOS compared to those treated with targeted therapy and immunotherapy.

**Table 1:** Treatment of NSCLC patients with EGFR exon 20.

Treatment Type (24)	Number of Patients	ORR (%)	mPFS (months)	mOS (months)
Platinum-Based Chemo	39	43	6.9	31.0
Conventional EGFR TKI	23	13	3.4	31.0
Single-Agent Immune Checkpoint Inhibitors	23	4	2.6	30.8

Amivantamab (JNJ-61186372) (7, 15, 25–27) is a bispecific antibody targeting EGFR and MET, exhibiting immune cell-directed activity. Due to its promising efficacy and safety profile, on May 21, 2021, the United States Food and Drug Administration (FDA) granted approval for its use in the treatment of EGFR-exon20ins in platinum-based chemotherapy-experienced or treatment-naïve patients with metastatic NSCLC (28, 29). Similarly, on September 15, 2021, the FDA also approved Mobocertinib for the treatment of locally advanced or metastatic NSCLC patients with EGFR exon 20 insertion mutations (28). In recent years, numerous clinical trials have made significant advancements in the treatment of NSCLC patients bearing EGFR exon 20 insertion mutations. This review aims to explore the status, recent advancements, and prospects in this field, The aim is to provide the latest clinical information and insights, establish robust clinical trial designs, propel the development of novel therapeutics, translate cutting-edge scientific research findings into clinical practice guidelines, and foster the creation of an effective treatment paradigm.

## 2 Methods for the detection of EGFR ex20ins

EGFR exon 20 insertion mutations (EGFR ex20ins), ranking as the third most common mutation, diverge from classical EGFR mutations by their influence on the kinase domain’s conformational dynamics rather than the ATP binding pocket crucial for kinase activity (21). The presence of exon 20 insertions induces an “αC-in” conformational shift in the α-C helix, thereby fostering constitutive activation and downstream signaling. Positioned distinctively outside the α-C helix, these mutations evade the adenosine triphosphate (ATP) binding pocket, typically exerting their effects through covalent binding to the ATP pocket and competitive inhibition of downstream signaling, rendering them refractory to conventional EGFR tyrosine kinase inhibitors (TKIs) save for the A763–764insFQEA mutation (30). Prior investigations have established that over 90% of insertion mutations occur within adjacent loops following the α-C helix within the intracellular domain of the receptor, with approximately 70% involving proximal loop insertions and 30% involving distal loop insertions, notably clustering between amino acids 766 and 775 and typically involving insertions or duplications of one to four amino acids (31). Despite the higher prevalence of proximal loop insertions, recent findings suggest that EGFR ex20ins patients harboring these mutations exhibit heightened responsiveness to novel targeted therapeutics such as Amivantamab and mobocertinib, attributed to the efficacy of insertions occurring within the C-helix segment against classical EGFR TKIs. Future endeavors necessitate further exploration of the sensitivity profiles of patients harboring proximal loop insertions to various targeted agents. Moreover, research into patients bearing less prevalent distal loop insertion mutations warrants attention. Tailoring interventions according to specific insertion mutation types holds promise for circumventing the limitations posed by chemotherapy and enhancing patients’ overall survival (OS) and progression-free survival (PFS).

EGFR exon 20 insertion mutations represent a frequently overlooked variant within the realm of EGFR mutations. The unequivocal diagnosis of EGFR exon 20 insertion mutations is pivotal for the treatment of non-small cell lung cancer (NSCLC) patients. Therefore, the development of accurate and efficient diagnostic tools becomes imperative. Next-generation sequencing (NGS) and polymerase chain reaction (PCR) are the primary methods employed for detecting EGFR mutations. However, PCR-based methods are limited to known mutation types and may potentially miss more than 50% of EGFR exon 20 insertions (32, 33), while NGS offers a greater likelihood of detecting the full spectrum of EGFR exon 20 insertions (33). Robichaux et al. demonstrated that, guided by next-generation sequencing (NGS) reports, structurally functional grouping can identify categories of drugs potentially effective against entire sets of mutations. This observation reflects the likelihood that mutations in different regions of the gene may induce similar changes in protein structure (34). A study conducted in the United States regarding EGFR testing patterns and the detection of EGFR exon 20 insertions has indicated a decline in the utilization of PCR-based tests and an increase in the adoption of NGS (19). Several alternative testing methods, such as Sanger sequencing and cobas sequencing, are available (35), but these techniques are not without inherent limitations. Sanger sequencing is designed to target specific pathogenic mutation sites, rendering it variable and less sensitive. In some instances, it may require up to 600 ng of DNA to detect EGFR mutations, a substantial challenge in many NSCLC patients. Furthermore, Sanger sequencing occasionally produces false-positive results, which can lead to unnecessary treatment and suboptimal therapeutic outcomes. In contrast, cobas sequencing requires smaller sample sizes and exhibits higher sensitivity compared to Sanger sequencing, but it is a more complex procedure demanding additional time and resources.

In summary, NGS presently stands as the gold standard method recommended by international guidelines for the diagnosis of late-stage NSCLC patients (28, 35–37). The ELEGANT study (38)((ClinicalTrials.gov registration number: NCT05737849), a nationwide, multi-center real-world registry study, is currently underway to investigate the testing methods, clinical-pathological characteristics, and molecular epidemiology of EGFR exon 20 insertion mutations in advanced non-small cell lung cancer patients in China. In order to achieve a more personalized and precise treatment approach, future research efforts should primarily focus on the following aspects (39, 40): (1) Understanding the impact of different insertion sites on the structure and function of the EGFR protein, thereby elucidating the pathogenic mechanisms of EGFR ex20ins and the efficacy of various drug treatments. This entails analyzing the structural and functional consequences of insertions within the EGFR protein, leading to insights into disease mechanisms and treatment responses. (2) Analyzing the molecular structures of various novel drugs to reveal their interactions with EGFR ex20ins at the molecular level. By elucidating the structural-activity relationship, identifying drug molecules with high selectivity and affinity for target mutations becomes possible. (3) Unique EGFR mutations drive distinct downstream signaling pathways. Characterization of downstream pathway activation specific to EGFR ex20ins may lead to the development of mutation-specific therapies targeting these pathways. Understanding the differences in downstream signaling pathways activated by different EGFR exon 20 insertion mutations compared to classical EGFR mutations is crucial for the development of therapies aimed at overcoming such differences.

## 3 Treatment of EGFR ex20ins

Multiple studies have confirmed that platinum-based chemotherapy remains the primary first-line treatment approach for patients with EGFR ex20ins mutations (24, 41, 42). The role of immune checkpoint inhibitors in these patients remains unclear, with suboptimal response rates and outcomes observed whether used alone or in combination with chemotherapy. Classical EGFR TKIs demonstrate limited clinical benefits in both first-line and ≥ second-line settings for such patients. Several novel TKIs targeting ex20ins are currently under development. Additionally, research by Ou et al. has shown that regardless of PD-L1 expression levels, patients receiving immune monotherapy in the first-line or later treatment lines exhibit low response rates (≤5%) and poor outcomes (OS: 6.1–8.9 months, PFS: 2.3–2.5 months), indicating ineffectiveness of immunotherapy in EGFR ex20ins and PD-L1-positive NSCLC patients (42). In the EXOTIC cohort, treatment regimens involving either single-agent chemotherapy or combination with immunotherapy constitute over half of the first-line treatment cases (43). This suggests that in the absence of approval for all novel targeted therapies, oncologists tend to manage exon20in NSCLC patients similarly to EGFR wild-type patients. In addition to describing the therapeutic effects of common treatments on EGFR ex20ins patients, Leal et al. also delineate the clinical-pathological characteristics of these mutations, highlighting variations in response to EGFR TKIs across different mutation sites (24).

As of present, the efficacy of chemotherapy has reached a plateau, prompting the exploration of promising drugs targeting unique, complex, and heterogeneous EGFR ex20ins mutations. Concurrently, novel and targeted combination therapy regimens are undergoing clinical trials in an effort to disrupt the current treatment landscape. It is hoped that these breakthroughs will alter the clinical management of patients, transitioning treatment paradigms from standard chemotherapy to the forefront of targeted therapy or other combinational approaches.

### 3.1 Previous treatment

#### 3.1.1 Chemotherapy

Currently, platinum-based doublet chemotherapy remains the standard treatment for EGFR exon 20 insertion (ex20ins) NSCLC patients. Wu et al. (20)(**Table 2**) observed that among 59 patients with EGFR ex20ins mutations, those who received first-line treatment with pembrolizumab (24 patients) exhibited superior progression-free survival (PFS) and overall survival (OS) compared to patients treated with first-line pemetrexed (7 patients) and docetaxel (10 patients). The PFS was 6.2 months versus 2.7 months, and OS was 28.0 months versus 15.4 months, with an objective response rate (ORR) of 29% and a disease control rate (DCR) of 75%. In a retrospective study from China (45)(**Table 2**) involving 119 stage IIIB/IV EGFR ex20ins NSCLC patients, 64.7% (77/119) received first-line chemotherapy, primarily with pemetrexed, and 13.45% (16/119) received second-line chemotherapy with pemetrexed. Patients who received first-line chemotherapy showed longer PFS compared to those who did not receive first-line chemotherapy (5.5 months vs. 3 months, P=0.0026), and their OS also tended to be longer (25.0 months vs. 19.6 months, P=0.0769).

**Table 2:** Studies of chemotherapy in patients with EGFR ex20ins NSCLC.

First-Line Treatment Type	Number of Patients	PFS (months)	OS (months)	ORR (%)	DCR (%)
Pembrolizumab (24) vs. Cisplatin-Based (7) vs. Gefitinib (10, 20)	59	6.2 vs. 2.7 vs. 3.4	28.0 vs. 15.4 vs. 31.0	29 vs. – vs. -	75 vs. – vs. -
First-Line Chemo (44) vs. Second-Line Chemo (16, 45)	119	5.5 vs. 3.0	25.0 vs. 15.6	–	–
Erlotinib Treatment vs. Chemotherapy (46)	46	3.0 vs. 26.0	26.0 vs. 31.0	–	–
First-Line Chemo vs. First-Line EGFR TKI vs. Second-Line Chemo (18)	165	6.4 vs. 2.9 vs. 4.0	–	–	–
Platinum-Based Double Chemo (47)	23	8.9	–	11.8	–

Naidoo et al. (46)(**Table 2**) conducted a study involving 46 EGFR ex20ins patients and 258 EGFR 19del/L858R patients. Following treatments with erlotinib, it was observed that the time to progression (TTP) in EGFR ex20ins patients was significantly shorter compared to EGFR 19del/L858R patients (3 months vs. 12 months, p<0.01). After chemotherapy, the median overall survival (OS) for EGFR ex20ins patients was also shorter than that of EGFR 19del/L858R patients (26 months vs. 31 months, p=0.53). It is evident that the majority of EGFR ex20ins patients do not benefit as substantially from EGFR TKI treatment, and chemotherapy appears to offer more favorable outcomes for EGFR ex20ins patients compared to EGFR TKI therapy.

Another study (18) (**Table 2**) involving 165 NSCLC patients with EGFR ex20ins mutations revealed that patients receiving first-line chemotherapy experienced longer progression-free survival (PFS) compared to those receiving full-dose EGFR TKI treatment (6.4 months vs. 2.9 months). Similarly, patients receiving first-line chemotherapy demonstrated a longer PFS compared to those receiving first-generation EGFR TKI treatment (6.4 months vs. 2.0 months). The objective response rate (ORR) for patients receiving first-line chemotherapy was 19.2%, with a 6-month disease control rate (DCR) of 41.3%. Patients receiving second-line chemotherapy also exhibited longer median PFS compared to those receiving second-line EGFR TKIs (4.0 months vs. 2.0 months). In a retrospective study led by Morita et al. (47) (**Table 2**), it was found that EGFR ex20ins NSCLC patients who received first-line platinum-based doublet chemotherapy (17 out of 23 patients) achieved an ORR of 11.8% and a median PFS of 8.9 months, both of which outperformed the efficacy of first-line EGFR TKIs and immune checkpoint inhibitors.

However, the efficacy of chemotherapy appears to have reached a plateau, underscoring the urgent need for novel therapies to further enhance the survival prospects of patients harboring such mutations (48).

#### 3.1.2 Immunotherapy

Immune checkpoint inhibitors have profoundly transformed the therapeutic landscape for NSCLC patients. However, based on previous research, it is evident that immune checkpoint inhibitors do not confer substantial benefits to NSCLC patients with classic EGFR mutations. To investigate whether immune checkpoint inhibitors yield similar results in the context of rare EGFR ex20ins mutations, Ji et al. (24) conducted a study involving 109 EGFR ex20ins NSCLC patients. Among them, 23 patients received single-agent immune checkpoint inhibitor therapy, achieving an objective response rate (ORR) of 4%, a median progression-free survival (mPFS) of 2.6 months, and a median overall survival (mOS) of 30.8 months. In comparison to NSCLC patients with classic EGFR mutations (38 patients), the EGFR ex20ins NSCLC patients (36 patients) treated with immune checkpoint inhibitors exhibited improved progression-free survival (PFS: 2.9 months vs. 1.9 months) and overall survival (OS: not reached vs. 11.5 months). Furthermore, they demonstrated better disease control rates (DCR) and ORR at 6 months and 12 months. These results suggest that, in certain scenarios, the use of immune checkpoint inhibitors in the treatment of EGFR ex20ins mutations may yield unexpected clinical benefits, warranting further exploration.

Patients with EGFR ex20ins in NSCLC exhibit lower tumor mutational burden (TMB), and high PD-L1 expression occurs in only a small fraction of patients. Metro et al. (49) (**Table 3**) observed that following first- or second-line treatment with immune checkpoint inhibitors, these patients experienced poorer median progression-free survival (mPFS: 1.6 months vs. 2.7 months) and median overall survival (mOS: 2.0 months vs. 8.1 months) compared to those who did not receive immune checkpoint inhibitors. Patients receiving immunotherapy had a shorter mOS (12.9 months vs. 25.2 months) when compared to patients not receiving immune therapy, indicating suboptimal efficacy of immunotherapy in this patient population.

**Table 3:** Studies of immunotherapy in patients with EGFR ex20ins NSCLC.

Treatment Type	Number of Patients	mPFS (months)	mOS (months)	ORR (%)
First-Line Immune Checkpoint Inhibitor vs. Second-Line Immune Checkpoint Inhibitor (49)	30	1.6 vs. 2.7	2.0 vs. 8.1	–
First-Line Chemo vs. Chemo + Immunotherapy (50)	122	No Difference	No Difference	–
First-Line Chemo vs. Chemo + Anti-angiogenesis (50)	122	5.93 vs. 7.73	–	18.2 vs. 38.1
Sintilimab + Anlotinib (51)	21	7.0	20.0	38.1

A clinical study conducted by Yang et al. (50) (**Table 3**) has revealed noteworthy findings in the context of the treatment of non-small cell lung cancer (NSCLC) patients with EGFR exon 20 insertions (EGFR ex20ins). The study encompassed 122 such patients who were administered first-line therapy, including chemotherapy alone, chemotherapy in combination with immunotherapy, and chemotherapy in conjunction with angiogenesis inhibitors. Despite the presence of PD-L1 expression or tumor mutational burden, the study did not observe a statistically significant difference in progression-free survival (PFS) between chemotherapy and chemotherapy combined with immunotherapy. However, a substantial disparity was observed between chemotherapy and chemotherapy combined with angiogenesis inhibitors in terms of objective response rate (ORR) (38.1% vs. 18.2%) and median PFS (7.73 months vs. 5.93 months). These results prompt a reconsideration of treatment strategies, akin to the clinical trials Impower 130 (52) and Impower 150 (53), where the combination of chemotherapy, immunotherapy, and anti-angiogenesis agents demonstrated potential benefits. In a prospective, single-arm, phase II trial (51) (**Table 3**), the efficacy and safety of the combination of sintilimab and anlotinib were investigated in rare EGFR-mutated NSCLC patients who had previously received second-line therapy. The results were based on a cohort of 21 patients, comprising 12 with EGFR exon 20 insertions and 8 with other rare mutations. The observed objective response rate (ORR) was 38.1%, with a disease control rate (DCR) of 85.7%. The median progression-free survival (PFS) reached 7.0 months, while the median overall survival (OS) was 20.0 months. The most common adverse event was hand-foot syndrome, occurring in approximately 9.5% of cases. Grade 3 adverse events were reported in 28.6% of patients, with no occurrences of grade 4 adverse events. In conclusion, this therapeutic combination exhibited promising efficacy and a favorable safety profile, underscoring the need for further investigation in future research endeavors. While several studies have reported inconsistent results regarding the response to immunotherapy, it would be premature to entirely discount the benefits it may offer. The emergence of novel combination therapy regimens may provide an additional avenue for improving patient outcomes, alongside the development of new therapeutic agents.

#### 3.1.3 Targeted therapy

On one hand, the presence of EGFR exon 20 insertion (EGFR ex20ins) mutations introduces an atypical amino acid configuration, forming a “wedge-like structure.” This structural anomaly significantly reduces the volume of the drug-binding pocket, consequently diminishing the binding region available for therapeutic agents. This, in turn, enhances steric hindrance, leading to decreased affinity with classic EGFR tyrosine kinase inhibitors (TKIs). On the other hand, EGFR ex20ins mutations exhibit a protein conformation akin to wild-type EGFR, displaying a binding affinity to ATP and an apparent similarity in binding affinities. This characteristic implies that effective drugs can not only inhibit EGFR ex20ins mutations but may also potentially target the wild-type EGFR protein. However, it is worth noting that certain studies have suggested that the A763–764insFQEA mutation remains sensitive to first, second, and third-generation EGFR TKIs.

As previously mentioned, a clinical study conducted by Wu et al. (20) involved 59 patients with EGFR exon 20 insertion (EGFR ex20ins) mutations. Among these patients, 16 individuals received first-line treatment with EGFR tyrosine kinase inhibitors (TKIs). Their observed objective response rate (ORR) was 6.3%. In comparison to first-line chemotherapy, the patients treated with first-line EGFR TKIs exhibited a less favorable median progression-free survival (mPFS) of 1.8 months versus 4.2 months. Notably, there was no significant difference in overall survival (OS) between the two groups.

##### 3.1.3.1 First, second, and third-generation EGFR TKIs

EGFR tyrosine kinase inhibitors (EGFR TKIs) have demonstrated remarkable efficacy in the treatment of non-small cell lung cancer (NSCLC) with the most common and effective mutations being EGFR 19del and L858R. However, EGFR exon 20 insertions (EGFR ex20ins), as the third most prevalent mutation, do not exhibit strong clinical benefits with EGFR TKIs. A study conducted by Popat et al. (54) (**Table 4**) reported findings from 29 EGFR ex20ins NSCLC patients. Among these, 10 patients who received first-generation EGFR TKI treatment exhibited a time to treatment failure (TTF) of 5.2 months and a median overall survival (OS) of 21.0 months. In contrast, 18 patients treated with afatinib had a TTF of 8.3 months and an OS of 22.5 months. In a subset of 23 EGFR ex20ins NSCLC patients, six patients who received first-generation EGFR TKI treatment displayed an objective response rate (ORR) of 16.7% and a duration of response (DOR) of 33%. Among 16 patients treated with afatinib, the ORR was 18.8%, and the DOR was 5.5%. Notably, outcomes for osimertinib were not evaluated due to the limited sample size. When compared to 20 patients who received first-line chemotherapy, patients treated with chemotherapy exhibited improved median time to treatment failure (mTTF) of 6.6 months and a superior ORR of 41%. These findings underscore the limited efficacy of EGFR TKIs in the context of EGFR ex20ins mutations, emphasizing the need for alternative therapeutic strategies in this subset of NSCLC patients.

**Table 4:** Studies of EGFR TKIS in patients with EGFR ex20ins NSCLC.

First-Line Treatment Type	Number of Patients	PFS (months)	OS (months)	ORR (%)	DCR (%)
First-generation EGFR TKI vs. Afatinib (54)	29	5.2 vs. 8.3	21.0 vs. 22.5	16.7 vs. 18.8	33 vs. 5.5

In the study previously referenced (18), a subgroup analysis was conducted within a cohort of 165 patients, where 23 patients with stage IV disease received first-line EGFR TKI treatment. Among these patients, 10 received gefitinib, 3 received icotinib, 1 received erlotinib, 3 received afatinib, and 6 received osimertinib. The observed objective response rate (ORR) in this subgroup was 8.7% (2 out of 23 patients), and the 6-month disease control rate (DCR) was also 8.7% (2 out of 23 patients). The median progression-free survival (mPFS) for these patients was 2.9 months. It is noteworthy that, as mentioned earlier, when compared to the targeted therapy group, the chemotherapy group demonstrated longer PFS. For patients who received first-line treatment with first-generation EGFR TKIs within this study, the ORR was 0%, and the mPFS was 2.0 months. Similarly, in comparison to the chemotherapy group, their PFS was notably shorter. This study collectively suggests that the efficacy of EGFR TKIs as first-line or second-line treatments for EGFR ex20ins NSCLC patients is limited. Furthermore, a retrospective study (55) indicated that, when compared to NSCLC patients with conventional EGFR mutations, EGFR ex20ins patients exhibited a shorter mPFS of 2.9 months following first-line treatment with EGFR TKIs, in contrast to the 10.5 months observed in the former group.

In a study aimed at assessing the efficacy of osimertinib in patients with EGFR exon 20 insertion (EGFR ex20ins) NSCLC (56), particularly those harboring the V769_D770insASV and D770_N771insSVD mutations, it was observed that EGFR ex20ins mutations share several signaling pathways and cellular biology characteristics with more common mutations. While osimertinib did not demonstrate superior efficacy compared to patients with common EGFR mutations, it exhibited superior anti-tumor activity when compared to erlotinib and afatinib. This suggests that osimertinib may offer greater clinical benefits for EGFR ex20ins NSCLC patients. Although osimertinib outperforms first and second-generation EGFR tyrosine kinase inhibitors (TKIs), it’s *in vitro* potency against EGFR ex20ins appears lower, resulting in comparatively reduced tumor growth inhibition. This suggests the potential need for higher drug concentrations in the clinical setting, such as a dose of 160 mg, which may reach the critical threshold for clinical efficacy. Piotrowska et al. reported the first clinical response to osimertinib in a patient with EGFR ins20 mutation (57). The patient, an 80-year-old female non-smoker diagnosed with metastatic lung cancer harboring EGFR ins20 S768_D770dup, underwent whole-brain radiation therapy for symptomatic metastatic brain lesions before opting for off-label high-dose (160 mg daily) osimertinib treatment. Subsequently, she experienced sustained extracranial partial response for 11 months. In phase II trials ECOG-ACRIN 5162 and POSITION20 (58, 59), moderate efficacy was observed with osimertinib 160 mg QD. Among previously treated Ex20Ins patients enrolled in these trials (n=20 and n=25, respectively), the ORR was 25% and 28%, with mPFS of 9.7 months and 6.8 months, respectively. Although demonstrating some potential anti-tumor activity, the 160 mg dose is not currently recommended as standard care, and results should be confirmed in phase III trials. Therefore, further clinical research is warranted to empirically determine whether osimertinib can provide clinical benefits for patients with rare EGFR ex20ins mutations and to address the dosage question effectively.

Given the suboptimal efficacy of monotherapy, the consideration of combination therapy may yield improved results. Research indicates that cetuximab monotherapy has little impact on the majority of EGFR exon 20 insertion (EGFR ex20ins) mutations. However, when cetuximab is combined with afatinib or osimertinib, it has demonstrated more effectiveness compared to monotherapy with afatinib or osimertinib. Notably, the combination of cetuximab with erlotinib has not yielded favorable results (60).

Furthermore, studies have suggested that resistance to EGFR TKIs can develop in EGFR ex20ins NSCLC patients, likely associated with the specific sequence of the insertion variant. Different insertion types may lead to distinct responses to EGFR TKI treatment. To gain a deeper understanding of the sensitivity of various insertion types to EGFR TKIs, further extensive efforts are required to explore and elucidate the nuances in treatment responses.

### 3.2 Emerging therapies and ongoing clinical studies

#### 3.2.1 Monoclonal antibody

##### 3.2.1.1 Amivantamab (JNJ-61186372)

Amivantamab is a novel, fully humanized IgG1 bispecific antibody targeting both EGFR and Met, with multiple mechanisms of action for treating EGFR and Met-driven diseases (7, 15, 25–27, 55, 61). It inhibits ligand-induced activation by blocking ligand-receptor binding, degrades receptor-antibody complexes to render them inactive, and induces Fc-effector molecule-mediated cytotoxicity for tumor cell elimination (21). Yun et al. demonstrated that Amivantamab exhibits clear anti-tumor activity in several Ba/F3 and PDC models expressing various EGFR ex20ins mutations. The compound effectively inhibits downstream signaling pathways and engages apoptosis mechanisms, impacting cell cycle progression and programmed cell death, ultimately restraining tumor cell proliferation.

The CHRYSALIS trial is a Phase I open-label dose-escalation clinical study (15). This study confirms that Amivantamab exhibits efficacy in both proximal and distal loop mutations. However, mutations closely following the proximal loop (codons 767–771) show a higher response rate compared to those located in the distal loop (codons 771–775).In the first part of the experiment (25), 25 EGFR ex20ins NSCLC patients, who had previously received platinum-based therapy, were treated with escalating doses of Amivantamab (140 mg in 3 patients, 350 mg in 3 patients, 700 mg in 9 patients, 1050 mg in 7 patients, and 1400 mg in 3 patients). No dose-limiting toxicities were observed at any dose level. Linear pharmacokinetics were evident at doses ≥350 mg, and steady-state concentrations reached or exceeded the preclinically established therapeutic target levels at doses ≥700 mg. Infusion-related reactions were the most common adverse events, occurring in approximately 76% of patients, with severity grades ≤2, primarily observed during the first administration. The second most common adverse event was rash/acneiform dermatitis, affecting about 40% of patients, with 16% of them experiencing grade 2 severity. Other related adverse events included dyspnea (24%), paronychia (24%), pruritus (20%), fatigue (20%), nausea (20%), and peripheral edema (cMET-related toxicity, 12%). Those data suggest that Amivantamab exhibits a favorable safety profile and substantial efficacy, making it a potential treatment option. These findings supported the recommended dose for the second part of the trial (15), in which 1050 mg (for patients with a body weight <80 kg) or 1400 mg (for patients with a body weight ≥80 kg) of Amivantamab was administered once weekly for the first month, and subsequently, every two weeks. In a cohort of 81 patients, the observed ORR was 40% (3 complete responses), the median duration of response (mDOR) was 11.1 months, and the median progression-free survival (mPFS) was 8.3 months. In a safety cohort of 114 patients, the most common adverse event was rash, affecting approximately 86% (98 patients), followed by infusion-related reactions (66%, 75 patients), and paronychia (45%, 51 patients). Grade 3 to 4 adverse events were most commonly low potassium levels (5%, 6 patients), followed by rash, pulmonary embolism, diarrhea, and neutropenia, each accounting for approximately 4% (1 patient each). Dose reduction occurred in 13% of patients during treatment, and 4% of patients discontinued treatment. In summary, Amivantamab appears to have manageable safety profiles and offers substantial efficacy through its dual-target mechanism. On May 21, 2021, the U.S. Food and Drug Administration approved its use for treating metastatic EGFR exon 20 insertion-positive NSCLC patients who have received prior platinum-based chemotherapy or are currently under treatment.

Research on Amivantamab extends far beyond the mentioned studies, with numerous clinical investigations currently underway to explore its deeper potential and efficacy in various EGFR mutation scenarios. The prospect of combination therapies involving Amivantamab is a promising area for further exploration. In September 2023, a Phase I multicenter, open-label study (25) (ClinicalTrials.gov registration number: NCT02609776) is designed to further investigate the recommended dosages, safety, and tolerability of Amivantamab as monotherapy, Amivantamab in combination with lazertinib, and Amivantamab combined with platinum-based chemotherapy in patients with EGFR ex20ins NSCLC. Amivantamab in combination with lazertinib has shown promising results in EGFR-mutated advanced non-small cell lung cancer that has recurred after osimertinib treatment. The safety profile of the combination therapy appears consistent with that of monotherapy and demonstrates substantial anti-tumor activity. Additionally, an ongoing Phase III PAPILLON study (62) (ClinicalTrials.gov registration number: NCT04538664) aims to evaluate the efficacy of Amivantamab in combination with platinum-based chemotherapy compared to platinum-based chemotherapy alone in EGFR ex20ins NSCLC patients. Preliminary results suggest an improved median progression-free survival (mPFS) for Amivantamab in combination with platinum-based chemotherapy compared to chemotherapy alone. Several other clinical trials are also in progress, including the Phase III MARIPOSA-2 study (63) (ClinicalTrials.gov registration number: NCT04988295) and Chrysis -2 (64) (ClinicalTrials.gov registration number: NCT04077463), among others. These ongoing studies are poised to provide further insights into the potential of Amivantamab in the treatment of EGFR ex20ins NSCLC. Given its specific targeting sites and impact on downstream signaling pathways, Amivantamab demonstrates not only promising efficacy in second-line therapy but also holds potential to surpass chemotherapy as a frontline treatment for patients with EGFR ex20ins mutations in the future (65) (**Table 5**).

**Table 5:** Ongoing clinical trials of monoclonal antibody targeted therapies.

Drug	Registration Number	Clinical Phase	Number of Patients	Comparative Therapies
Amivantamab	NCT02609776	I	780	Amivantamab vs. Lazertinib Treatment
Amivantamab	NCT04538664	III	308	Amivantamab in Combination with Platinum-Based Chemotherapy vs. Monotherapy
Amivantamab	NCT04988295	III	776	Amivantamab in Combination with Lazertinib and Platinum-Based Chemotherapy vs. Monotherapy
Amivantamab	NCT04077463	I	460	Amivantamab in Combination with Lazertinib vs. Monotherapy with Lazertinib

#### 3.2.2 Small molecule TKI

##### 3.2.2.1 Mobocertinib

Mobocertinib (TAK-788) is a novel and highly selective irreversible TKI (Tyrosine Kinase Inhibitor) targeting EGFR ex20ins/HER2. This irreversible binding mechanism enhances efficacy through higher affinity binding, prolonged inhibition of EGFR kinase activity, and greater overall selectivity. Its isopropyl ester moiety targets proteins near the α C-helix, an unutilized binding site by osimertinib. The isopropyl ester of Mobocertinib is specifically designed to interact with residues in this pocket, exploring subtle conformational differences between EGFR ex20ins mutants and WT EGFR. Gonzalvez et al. reported the selective activity of TAK-788 against 14 different EGFR exon 20 insertion mutants expressed in Ba/F3 cell line models, effectively inhibiting all tested forms of activated EGFR and HER2 (66). It has been evaluated in a Phase I/II trial(ClinicalTrials.gov identifier NCT02716116) (67). In the Phase I dose-escalation trial, 101 patients who had previously received two or more cancer therapies were enrolled, and they were administered Mobocertinib at doses ranging from 5 to 180 mg/day. The final dose determined for the Phase II expansion was 160 mg/day. Among the 28 refractory patients included in the study, those treated with Mobocertinib (160 mg/day) showed an objective response rate (ORR) of 43%, a median progression-free survival (mPFS) of 7.3 months, a median duration of response (mDOR) of 13.9 months, and a disease control rate (DCR) of 86%. Among the 72 patients treated with Mobocertinib (160 mg/day), the most common adverse events included diarrhea, nausea, and rash, accounting for approximately 40%. A total of 25% of patients required dose reductions, and 14% of patients discontinued treatment. At the 2022 European Society for Medical Oncology (ESMO) Congress (68), results from an open-label non-randomized Phase I/II clinical trial assessing the efficacy and safety of Mobocertinib in previously platinum-treated EGFR ex20ins metastatic NSCLC patients were presented. In this experiment, the most common EGFR ex20ins mutations observed were V769_D770insASV, D770_N771insSVD, and H773_V774insNPH, with 74% of patients exhibiting proximal loop insertions (at positions 767–772) and 25% displaying distal loop insertions (at positions 773–775), indicating that drug sensitivity is associated with the insertion site. In the cohort of 114 patients with a history of platinum-based chemotherapy (PPP cohort), the objective response rate (ORR) assessed by the Independent Review Committee (IRC) was 28%, the disease control rate (DCR) was 78%, the median duration of response (mDoR) was 17.5 months, and the median progression-free survival (mPFS) was 7.3 months. The median overall survival (mOS) was 24.0 months. The investigator-assessed ORR was 35%. Results from the 96-patient EXCLAIM extension cohort were like the PPP cohort, with an IRC-confirmed ORR of 25% and an investigator-confirmed ORR of 32%.

On September 15, 2021, the U.S. Food and Drug Administration (FDA) approved Mobocertinib for the treatment of locally advanced or metastatic NSCLC patients with EGFR ex20ins mutations. In January 2023, Mobocertinib was granted approval for marketing in China, primarily for the treatment of EGFR ex20ins NSCLC patients who have experienced disease progression during or after platinum-based chemotherapy. However, as with previous generations of EGFR TKIs, acquired resistance during treatment with these TKIs still occurs. Hamada et al. demonstrated through experiments that the C797S secondary mutation in insFQEA and insSVD is the cause of acquired resistance to all mobocertinib. However, in other 20ins mutations (insASV, insNPH, and insH), secondary mutations such as T790M or C797S contribute to acquired resistance to mobocertinib. Interestingly, Sunvozertinib exhibits good activity against T790M-resistant cells. Erlotinib shows activity against insFQEA with the C797S mutation (69). Based on previous research findings, Mobocertinib demonstrated significantly higher efficacy compared to standard chemotherapy regimens. To further validate these observations, a Phase III clinical trial, known as the EXCLAIM-2 study (70)(**Table 6**)(ClinicalTrials.gov identifier NCT04129502), was conducted to evaluate the efficacy of Mobocertinib compared to platinum-based doublet chemotherapy in treatment-naive patients. Approximately 318 patients were enrolled in the study and randomly assigned to either receive oral Mobocertinib or platinum-based chemotherapy. However, the trial was ultimately terminated due to the lack of observed clinical benefit. Subsequently, on October 2, 2023, Takeda Pharmaceuticals voluntarily withdrew Mobocertinib from the U.S. market and planned a global voluntary withdrawal. This decision reflects the challenges faced in achieving anticipated clinical benefits in treatment-naive patients.

**Table 6:** Ongoing clinical trials of small-molecule TKI targeted therapies.

Drug	Registration Number	Clinical Phase	Number of Patients	Comparative Therapies
Mobocertinib	NCT04129502	III	354	Mobocertinib vs. Platinum-Based Chemotherapy
Mobocertinib	NCT05863819	–	120	Mobocertinib
Mobocertinib	NCT02716116	I/II	334	Mobocertinib
Poziotinib	NCT03318939	II	603	Poziotinib
Poziotinib	NCT03066206	II	116	Poziotinib
Furmonertinib	NCT05465343	II	36	Furmonertinib
Furmonertinib	NCT05379803	II	40	Furmonertinib
Furmonertinib	NCT05364073	I	170	Furmonertinib
Furmonertinib	NCT05466149	II	40	Furmonertinib
Furmonertinib	NCT04858958	Ib	30	Furmonertinib
Furmonertinib	NCT05607550	III	375	Furmonertinib vs. Platinum-Based Chemotherapy
DZD9008	NCT05668988	III	320	DZD9008 vs. Platinum-Based Chemotherapy
DZD9008	NCT03974022	I/II	326	DZD9008
DZD9008	NCT05559645	Not Applicable	110	DZD9008
DZD9008	NCT05712902	II	104	DZD9008
Zipalertinib	NCT05967689	II	160	Zipalertinib
Zipalertinib	NCT04036682	I/II	284	Zipalertinib
Zipalertinib	NCT05973773	III	312	Zipalertinib in Combination with Chemotherapy vs. Monotherapy
JMT101	NCT05132777	II	155	JMT101 in Combination with Osimertinib Treatment
FWD1509	NCT05068024	I/II	30	FWD1509
YK029A	NCT05767866	I/II	160	YK029A

##### 3.2.2.2 Poziotinib

Poziotinib is an irreversible inhibitor targeting EGFR/HER2/HER4. It has the capacity to inhibit classic EGFR mutations, T790M mutations, and high HER2 expression (19, 71). Previously, it was reported that EGFR ex20ins has a sterically hindered drug-binding pocket, but the size and flexibility of poziotinib enable it to overcome this challenge. This could be attributed to its unique molecular characteristics. Poziotinib is centered on a less rigid quinazoline core, akin to second-generation EGFR inhibitors. Additionally, poziotinib features small terminal and substituent linking groups, rendering it more compact and flexible compared to current second-generation and third-generation inhibitors. Based on these features, three-dimensional modeling predicts that poziotinib can tightly bind to the EGFR ex20ins binding pocket and may also be effective against structurally analogous exon 20 insertions in HER2 (72). In Ba/F3 cell models, Poziotinib exhibits the lowest average IC50 value (73). Elamin et al. observed that the sensitivity of poziotinib is highly dependent on the insertion site, with proximal loop insertions (amino acids A767 to P772) being more sensitive compared to distal loop insertions (34). Clinical confirmation of this phenomenon revealed observed objective response rates (ORR) of 46% and 0% in proximal and distal loop insertions, respectively (p=0.0015), thus establishing poziotinib as an effective inhibitor for these mutations. Notably, in EGFR 19Del and EGFR ex20ins, Poziotinib demonstrates significantly superior efficacy compared to drugs like Afatinib and Neratinib. Its potency is 40 times higher than that of Afatinib and 100 times stronger than Osimertinib.

In a Phase II clinical trial (74) involving 40 patients treated with Poziotinib, the 8-week objective response rate (ORR) was 58%, and the disease control rate (DCR) was 83%. Of the patients, 65.1% had received at least second-line treatment, and 60% experienced adverse events of grade 3 or higher. Dose reductions were observed in 45% of patients to 12mg and 17.5% of patients to 8mg. The most common adverse event was rash, occurring in approximately 27.5% of patients, with treatment discontinuation in one case due to grade 3 rash. Diarrhea was the second most common adverse event, affecting about 12.5% of patients. These results suggest that Poziotinib offers controlled safety and a certain degree of efficacy. To further validate its reliability, a multicenter ZENITH20 study, Phase II (75), delved into the effectiveness of Poziotinib in previously treated EGFR ex20ins NSCLC patients. In this study, which included 90 patients who had undergone second-line treatment, the ORR was approximately 27.8%, the DCR was about 70%, median progression-free survival (mPFS) was 5.5 months, and median duration of response (mDOR) was 5.1 months. The primary adverse events observed were rash (48.9%), diarrhea (25.6%), and stomatitis (24.4%). A total of 76.7% of patients required a reduction in treatment dosage, and 13.3% discontinued treatment. The results of this trial differed from previous studies, revealing limited safety and clinical efficacy. Consequently, the U.S. Food and Drug Administration denied Poziotinib’s approval for marketing, affecting subsequent research efforts (**Table 6**).

In a Phase II multicohort multicenter ZENITH20-C4 study (44), 80 untreated NSCLC patients with EGFR ex20ins were enrolled. Patients received oral Poziotinib at 16mg QD (47 patients) or 8mg BID (33 patients). The ORR was 39%, and the DCR was 73%, with a median mDOR of 5.7 months and mPFS of 5.6 months. In this trial, 80% of patients experienced tumor shrinkage. The most common grade 3 treatment-related adverse events were rash (QD, 45%; BID, 39%), stomatitis (QD, 21%; BID, 15%), and diarrhea (QD, 15%; BID, 21%). These findings suggest that Poziotinib has a certain degree of safety and efficacy for previously untreated EGFR ex20ins NSCLC patients. However, inevitably, treatment leads to the development of resistance. A study employing ENU mutagenesis screening with various EGFR ex20 insertion variants (A763insFQEA, V769insASV, D770insSVD, and H773insNPH) confirmed EGFR C797S as a potential mediator of resistance to poziotinib (76). Furthermore, it further demonstrated that EGFR T790M mutation also confers *in vitro* resistance to poziotinib. However, the future application of Poziotinib requires further exploration to develop safer and more effective treatment strategies.

##### 3.2.2.3 Furmonertinib (AST2818)

Furmonertinib, a domestically developed third-generation EGFR TKI inhibitor, received regulatory approval in China in March 2021 for the treatment of NSCLC patients with EGFR T790M mutations. The chemical structure of furmonertinib closely resembles that of osimertinib (77, 78). However, unlike osimertinib, furmonertinib introduces a strongly hydrophobic trifluoroethoxy pyridine moiety. This enables its binding to the hydrophobic pocket of the ATP-binding region, comprised of residues such as L792 and M793. This alteration not only enhances furmonertinib’s binding affinity to EGFR and its kinase selectivity but also improves its metabolic properties. The efficacy of furmonertinib is independent of the location of EGFR ex20ins mutations (79), it has demonstrated efficacy not only in patients with classic EGFR mutations but also in those with EGFR ex20ins mutations. In BaF3 cell models, the average IC50 for Furmonertinib ranges from 11 to 20nM. Preclinical studies have demonstrated efficacy in treating patients with EGFR ex20ins as well as those with central nervous system involvement (78). The preliminary results of the Phase Ib FAVOUR 1 study (80) (ClinicalTrials.gov registration number: NCT04858958) were presented at the 2021 ESMO conference. The study aimed to evaluate the clinical efficacy and safety of Furmonertinib. Cohort 1 included ten treatment-naïve patients receiving Furmonertinib 240mg QD, while cohorts 2 and 3 comprised previously treated patients randomly assigned to receive 240mg QD and 160mg QD, respectively. In cohort 1, all patients remained under treatment. At the data cutoff, the objective response rate (ORR) was 5/7 (all partial responses, with 2 cases pending confirmation). Three patients exhibited stable disease. The independent review committee (IRC) assessed the confirmed ORR (cORR) as 60%, while the investigator (INV) assessment reported a cORR of 70%. All patients experienced a reduction in target lesions. The most common adverse reactions included diarrhea, paronychia, and skin fissures (each accounting for 30%), and no adverse events of grade 3 or higher were observed. No dose reductions or treatment discontinuations were reported. This study indicated that Furmonertinib demonstrated preliminary superior safety and effectiveness.

A retrospective single-arm analysis presented at ESMO in 2023 revealed promising results. Among 20 patients with EGFR ex20ins mutations, Furmonertinib treatment led to 14 cases of partial response and 6 cases of stable disease. The median progression-free survival (mPFS) was 10.2 months, and the median duration of response (mDOR) was 8.5 months. All patients showed a reduction in target lesions, and no grade 3 or higher adverse events were observed. In comparison to treatment with Osimertinib, Furmonertinib demonstrated longer mPFS and mOS (median overall survival). Furmonertinib exhibited the highest binding activity to EGFR ex20ins when compared to other EGFR TKIs such as Erlotinib and Gefitinib. This study further confirmed the effectiveness of Furmonertinib and suggests that it holds promise as a treatment strategy for late-stage EGFR ex20ins NSCLC patients.

In another retrospective study aimed at evaluating the efficacy and safety of Furmonertinib, 53 advanced NSCLC patients with EGFR ex20ins mutations were included (81). The study reported an ORR of 37.7% and a disease control rate (DCR) of 92.5%. The 6-month PFS rate was 69.4%. The most common adverse events were diarrhea and rash (both at 26.4%), with no observed grade 3 or higher adverse reactions. In summary, Furmonertinib demonstrates good safety, even at a dose of 240mg, with no dose-dependent toxicity. However, given the limited number of patients in clinical studies of Furmonertinib, potential biases exist, necessitating further validation of its efficacy with larger patient cohorts in the future (**Table 6**).

##### 3.2.2.4 Sunvozertinib (DZD9008)

Sunvozertinib is a novel, irreversible EGFR/HER2 inhibitor. On January 31, 2022, the U.S. Food and Drug Administration granted Breakthrough Therapy Designation (BTD) to Sunvozertinib, making it the only first-line drug to receive dual BTD status in both China and the United States. It is primarily intended for the treatment of locally advanced or metastatic NSCLC patients with EGFR ex20ins mutations who have progressed after platinum-based chemotherapy or after disease progression (82).

Clinical studies have demonstrated that Sunvozertinib’s tumor-inhibitory activity is independent of the EGFR ex20ins mutation site. Sunvozertinib exhibits favorable pharmacokinetic/pharmacodynamic correlations in xenograft models but shows weak activity against wild-type EGFR. In Ba/F3 cell lines, the IC50 of Sunvozertinib ranges from 6 to 40 nmol/L, displaying potent activity in downregulating pEGFR. The structure of Sunvozertinib differs from Mobocertinib, with an open C-5 position on the pyrimidine (proximal to Thr790), and a more flexible phenylamino moiety substituted at the pyrimidine C-4 position instead of the less flexible methyl indole on Osimertinib, aiming to flexibly accommodate different mutations with slight variations in the size of the ATP binding pocket. Through specific interactions with the adjacent C-helix and P-loop below, optimization of the pyrimidine tethering moiety at C-4 and solvent-exposed amino-terminal groups counteracts rare EGFR ex20ins (82).

Two ongoing Phase I studies (83) (**Table 6**), WU-KONG1 (Phase I/II, multi-national, ClinicalTrials.gov registration number: NCT03974022) and WU-KONG2 (Phase II, China, China Drug Trial Registration number: CTR20192097), are currently investigating Sunvozertinib. These studies have enrolled recurrent NSCLC patients with EGFR mutations, including EGFR ex20ins and HER2 mutations, who have previously received standard treatments. A total of 102 patients (54 from WU-KONG1 and 48 from WU-KONG2) have been treated with Sunvozertinib, of which 62 had EGFR ex20ins mutations. As of June 2022, among the 56 evaluable patients, the best objective response rate (ORR) across all doses was 41.1%, with a confirmed ORR of 37.5%. At the recommended Phase 2 doses of 200mg, the ORR was 45.5%, and at 300mg, the confirmed ORR was 41.9%. In the dose-expansion cohort (200–400mg), the best ORR was 47.4%, with a confirmed ORR of 44.7%. The median follow-up was 4.2 months, and median duration of response (mDoR) exceeded 3.5 months. Median progression-free survival (PFS) was more than 4 months, with neither reaching their respective medians. The longest DoR extended beyond 8 months, with 65.2% of 23 patients still on ongoing treatment with a response. The study suggests that first-line Sunvozertinib treatment has shown preliminary efficacy in EGFR ex20ins patients. Furthermore, preliminary anti-tumor activity was observed in patients with EGFR sensitizing mutations, EGFR sensitizing/T790M double mutations, and HER2 exon20ins mutations.

In addition, at the 2023 ASCO, data from a Phase II study, WU-KONG 6 (84)(**Table 6**) (ClinicalTrials.gov registration number: NCT05712902 and CTR20211009), were presented. This study included 97 EGFR ex20ins NSCLC patients who had previously progressed after chemotherapy and received at least one treatment. At a dose of 300mg QD, the ORR was 60.8%, disease control rate (DCR) was 87.6%, with the longest duration of response (DOR) exceeding 11 months, and approximately 90% of target lesions showed reduction. Similar results were observed in patients with brain metastases and those who developed resistance after Amivantamab treatment. The study by Hamada et al. (69) suggests that Sunvozertinib may become the preferred treatment for patients who develop resistance after Mobocertinib therapy. These results further confirm the favorable clinical activity and safety of Sunvozertinib, making it superior to current treatments for EGFR ex20ins NSCLC patients.

The ongoing clinical trial WU-KONG 28 (85) (**Table 6**) (Phase III, multi-national, ClinicalTrials.gov registration number: NCT05668988) aims to compare first-line Sunvozertinib with chemotherapy to further explore the safety and clinical efficacy of Sunvozertinib.

##### 3.2.2.5 Zipalertinib (CLN-081, TAS6417)

Zipalertinib is an irreversible EGFR tyrosine kinase inhibitor (TKI) that selectively targets cells with EGFR ex20ins mutations, exhibiting potent inhibitory effects against this mutation. Zipalertinib’s structure tightly fits the ATP binding pocket of EGFR ex20ins kinase and covalently modifies the residue containing Cys797 (86). In xenograft models, Zipalertinib inhibits EGFR phosphorylation to block the PI3K-AKT and RAS-MAPK signaling pathways, ultimately resulting in tumor regression. The efficacy and selectivity of Zipalertinib have been demonstrated *in vitro* models using NIH/3T3 and Ba/F3 cell lines containing various EGFR ex20 ins mutations. Zipalertinib exhibited significantly higher potency against mutant variants compared to wild-type EGFR, with IC50 ratios of 134-fold for A763_Y764insFQEA, 134-fold for D770_N771insSVD, 174-fold for D770_N771insG, 6.37-fold for V769_D770insASV, 4.55-fold for H773_V774insPH, and 4.51-fold for H773_V774insNPH (86). On January 4, 2022, the U.S. Food and Drug Administration granted it Breakthrough Therapy Designation (BTD) for the treatment of locally advanced or metastatic NSCLC patients with EGFR ex20ins mutations who have experienced disease progression after platinum-based chemotherapy.

In a Phase I/IIa study (87), patients with recurrent or metastatic NSCLC with EGFR ex20ins mutations who had previously received platinum-based chemotherapy were enrolled. These patients were administered Zipalertinib at oral doses ranging from 30–150mg, twice daily (BID). Objective responses were observed at all dosages, with 54 patients experiencing target lesion reduction after six weeks of treatment. Among the patients, 33% achieved partial responses (PR), and 59% achieved stable disease (SD). The median duration of response (mDOR) for all dosage levels in the 73 patients was 10 months. A confirmed PR was seen in 38.4% (28 patients), and the disease control rate (DCR) was 95.9%, with a median progression-free survival (mPFS) of 10 months.In patients treated with 100mg BID or ≤65mg BID of Zipalertinib, mDOR had not been reached by the data cutoff, with mPFS of 12 months and 8 months, respectively. The most common adverse event across all dosage levels was rash, occurring in approximately 80% of patients, with only two patients experiencing grade 3 rash at the highest dosage level. Diarrhea was the second most common side effect, seen in approximately 30% of patients, with only one patient experiencing grade 3 diarrhea at the 150mg dosage level. A 14% dose reduction and 8% treatment discontinuation were reported. This study indicates that proximal loop mutations are the most common subtype of mutations, followed by distal loop mutations and helical domain mutations, with ORR rates of 41.5%, 22%, and 0%, respectively. This study suggests that Zipalertinib demonstrates a favorable ORR and PFS in the treatment of NSCLC patients with EGFR ex20ins mutations, with manageable adverse events, making it a potential alternative for patients who have undergone multiple lines of treatment. However, over time, some patients inevitably develop resistance to Zipalertinib. Kagawa et al. demonstrated that similar to Poziotinib, all Zipalertinib-resistant clones harbored the EGFR C797S mutation (88). Ba/F3 cells carrying C797S (Ba/F3-C797S) exhibited resistance to EGFR tyrosine kinase inhibitors. Interestingly, a potential small molecule inhibitor, Pimitespib (selective heat shock protein 90), was found to overcome CLN-081 resistance. Jorge et al. suggest that clinical development of a certain type of Hsp90 inhibitor, either alone or in combination with other therapies, for the management of EGFR and/or ERBB2 mutant NSCLC in TKI-resistant or TKI-resistant environments, including tumors harboring EGFR ex20ins mutations, may yield unexpected therapeutic efficacy.

Currently, a Phase III global, multicenter study called REZILIENT 3 (89) (ClinicalTrials.gov registration number: NCT05973773) is underway to investigate the efficacy and safety of Zipalertinib in combination with chemotherapy compared to chemotherapy alone in EGFR ex20ins patients. Data from this study have not yet been publicly disclosed. The research on Zipalertinib holds great promise for the future, offering numerous possibilities for further exploration (**Table 6**).

##### 3.2.2.6 JMT-101

JMT101 is a recombinant fully humanized monoclonal antibody targeting the epidermal growth factor receptor (EGFR), similar to cetuximab but with six times greater affinity for the receptor (90). Previous research has indicated that cetuximab in combination with afatinib or osimertinib can inhibit the activity of EGFR exon 20 insertion (EGFR ex20ins) (60). As part of a Phase Ib clinical trial (90), the safety, tolerability, and anti-tumor activity of JMT101 in combination with afatinib or osimertinib were explored. In Ba/F3 cells, JMT101 alone at doses ranging from 1–200 μg/L had minimal efficacy. However, when combined with afatinib or osimertinib, it demonstrated potent anti-proliferative effects. In a xenograft model, JMT101 led to a 60% tumor growth inhibition, suggesting that the anti-tumor activity of JMT101 may require the involvement of effector cells. In this study, the dose expansion phase involved JMT101 at 6 mg/kg and osimertinib at 160 mg. Among 121 efficacy-evaluable patients, the investigator-assessed confirmed objective response rate (ORR) was 36.4%, and the disease control rate (DCR) was 95.0%. The median progression-free survival (mPFS) was 8.2 months, with a tumor shrinkage rate of 91.7%. In a subgroup of 53 heavily treated NSCLC patients who had progressed after platinum-based chemotherapy and received JMT101 in the second-line setting, the confirmed ORR was 34.0%, the median duration of response (mDOR) was 13.3 months, and the mPFS was 9.2 months.In this study, 62 patients had brain metastases, with 80.6% of them being previously untreated. Among 16 patients with brain metastases, 13 (81.3%) exhibited reductions in brain tumors. The intracranial DCR was 87.5%, and the investigator-assessed intracranial ORR was 25.0%. Across all doses, the most common adverse event was rash, affecting approximately 76.9% of patients, followed by diarrhea, which was observed in about 63.6% of patients. In patients with helical, proximal loop, and distal loop insertions, the confirmed objective response rates were 75% (3/4, 95% CI = 19.4–99.4), 36.7% (36/98, 95% CI = 27.2–47.1), and 28.6% (4/14, 95% CI = 8.4–58.1), respectively. This study demonstrates that the dual-targeting approach of JMT101 in combination with afatinib or osimertinib exhibits good safety and anti-tumor activity. It also shows promise in previously untreated patients with brain metastases, potentially offering a new treatment strategy in the future.

Currently, an ongoing Phase II trial (91)(**Table 6**)(ClinicalTrials.gov registration number: NCT05132777) further explores the safety and efficacy of JMT101 in combination with osimertinib for the treatment of advanced EGFR exon 20 insertion-positive NSCLC patients.

##### 3.2.2.7 FWD1509

FWD1509 is an irreversible EGFR tyrosine kinase inhibitor (TKI) that exhibits higher targeting activity against EGFR ex20ins, classic EGFR mutations, and T790M mutations compared to wild-type EGFR. Additionally, FWD1509 has demonstrated clinical activity in patients with brain metastases. In various preclinical studies, FWD1509 has shown favorable safety profiles and significant inhibition of EGFR ex20ins, showcasing promising anti-tumor activity. In January 2021, FWD1509 received Investigational New Drug (IND) approval from the U.S. Food and Drug Administration, and in May 2021, it obtained clinical approval from the National Medical Products Administration (NMPA) in China. Currently, a Phase I/II clinical trial (92) (ClinicalTrials.gov registration number: NCT05068024) is ongoing to evaluate the safety and efficacy of FWD1509 in advanced NSCLC patients. This study aims to provide further insights into the potential of FWD1509 as a treatment option for patients with EGFR mutations and EGFR ex20ins, highlighting its clinical promise (**Table 6**).

##### 3.2.2.8 YK-029A

YK-029A is a third-generation EGFR tyrosine kinase inhibitor (TKI) developed by a national research team. In a multicenter Phase I clinical trial (93), 108 patients with EGFR ex20ins mutations were enrolled, with the primary focus on evaluating its safety and tolerability. During the dose-escalation phase, patients received varying doses ranging from 50–250mg per day, and in the dose-expansion phase, treatment-naïve patients were administered 200mg per day. Among the 26 evaluable patients, an impressive 73.1% objective response rate (ORR) and a 92.3% disease control rate (DCR) were observed. Notably, no dose-limiting toxicities were observed in these patients, indicating excellent tolerability of YK-029A. Common adverse events included anemia (50.9%), diarrhea (49.1%), and rash (34.3%).

For treatment-naïve patients with EGFR ex20ins non-small cell lung cancer (NSCLC), YK-029A demonstrated both favorable safety and notable anti-tumor activity. Currently, preparations are underway for a Phase II clinical trial of YK-029A (ClinicalTrials.gov registration number: NCT05767866) to further investigate its efficacy in this patient population (94) (**Table 6**).

##### 3.2.2.9 BEBT-109

BEBT109 is an effective, broad-spectrum, selective EGFR tyrosine kinase inhibitor (TKI) (95). Preclinical trial results indicate that BEBT109 is rapidly absorbed and cleared *in vivo*, significantly reducing the potential for drug accumulation and associated toxicity. Compared to osimertinib, BEBT109 demonstrates a significantly higher inhibitory potency against EGFR ex20ins mutations, approximately 4.1 times stronger. Currently, plans are underway for a Phase II study involving the treatment of non-small cell lung cancer (NSCLC) patients with EGFR ex20ins and other rare EGFR mutations using BEBT109. The objective of this study is to evaluate the efficacy and safety of BEBT109 in this patient population. This research holds promise as a potential therapeutic option for individuals with rare EGFR mutations (95) (**Table 6**).

##### 3.2.2.10 PLB-1004

PLB-1004 is a novel irreversible monoanilino-pyrimidine small molecule inhibitor with high selectivity for targeting classic EGFR mutations and T790M mutations.

A multicenter study conducted in China (96) aimed to assess the safety, tolerability, pharmacokinetics, anti-tumor effects, and determine the recommended Phase 2 dose (RP2D) for PLB-1004. The study involved dose escalation ranging from 10mg to 480mg administered once daily (QD). The primary focus during dose expansion was on the 320mg QD and 400mg QD levels. Among the enrolled patients, 38 carried EGFR ex20ins mutations. Of the 26 patients who underwent tumor assessment, an impressive 57.7% objective response rate (ORR) and a 100% disease control rate (DCR) were observed. Notably, 8 patients presented with baseline brain metastases, and 37.5% of them achieved partial responses (PR). The most common adverse events included diarrhea (approximately 75%), followed by rash (60%) and oral ulcers (43%), among others. Moreover, varying degrees of Grade 3 adverse events were reported. It’s important to note that no dose-limiting toxicities were observed across all dose groups. This Phase I study provided preliminary evidence of the efficacy and safety of PLB-1004, particularly demonstrating efficacy in patients with brain metastases and highlighting its robust anti-tumor activity. However, due to the occurrence of multiple adverse events, further research is required to determine the appropriate dosage or potential combination therapy strategies.

Currently, a Phase II Kannon study (97) (ClinicalTrials.gov registration number: NCT06015503) is ongoing to evaluate the safety and effectiveness of PLB-1004 in patients with late-stage and metastatic NSCLC carrying EGFR ex20ins mutations (**Table 7**).

**Table 7:** Ongoing clinical trials of novel drug therapies.

Drug	Registration Number	Clinical Phase	Number of Patients	Comparative Therapies
PLB-1004	NCT06015503	II	157	PLB-1004
BLU-451	NCT0521873	I/II	332	BLU-451 vs. BLU-451 in Combination with Platinum-Based Chemotherapy
BTDX1535	NCT05256290	I	120	BTDX1535
GB263T	NCT05332574	I/II	120	GB263T
BI 1810631	NCT04886804	II	371	BI 1810631
STX-721	NCT06043817	I/II	120	STX-721
BAY2927088	NCT05099172	I	340	BAY2927088

##### 3.2.2.11 Compound 1 (a, b, c), DS2087b

Compound 1 (a, b, c) is a pyrimidine-based benzylaminomethyl ester series known for its ability to bind to the Cys797 of EGFR and hydrophobic pockets (98). Jang et al. observed a hydrophobic pocket located at the backside of the ATP-binding site of EGFR, which did not bind to osimertinib. Utilizing the original pyrimidine core, a series of novel compound analogs were generated by incorporating substituents designed to interact with EGFR within the deep hydrophobic pocket. While its efficacy is lower than that of Poziotinib, Compound 1a exhibits anti-proliferative activity against EGFR ex20ins, surpassing currently approved second and third-generation EGFR TKIs. It retains inhibitory effects against EGFR and HER2 ex20ins mutations. However, its clinical acceptance has been limited due to factors such as low bioavailability, high clearance rates, and short half-life. DS2087b, like Poziotinib, also serves as a selective inhibitor for EGFR and HER2 ex20ins mutations.

##### 3.2.2.12 BLU-451 (LNG-451)

BLU-451, represents the first covalent inhibitor within its class. It possesses several advantageous characteristics, including central nervous system penetration and selectivity for wild-type EGFR. LNG-451 exhibits a therapeutic efficacy like Mobocertinib and, in certain aspects, surpasses that of osimertinib. Notably, it demonstrates exceptional therapeutic outcomes in patients with brain metastases. Currently, an ongoing Phase I/II clinical trial (99) (ClinicalTrials.gov registration number: NCT0521873) is actively assessing BLU-451 to further evaluate its efficacy and safety (**Table 7**).

Furthermore, several new clinical drugs, including APL-1898, BDTX-189, BDTX-1535, HTMC-0503, GB263T, BI 1810631, STX-721, BAY2927088, and related clinical studies, are in active development. These endeavors aim to provide additional treatment options for cancer patients, thereby contributing to the advancement of oncology therapies (**Table 7**).

## 4 Discussion

EGFR ex20ins, as the third most common EGFR mutation, holds a significant place within the broad spectrum of lung cancer patients. In comparison to the more well-studied EGFR19del and L858R mutations, research, and clinical data on EGFR ex20ins are relatively limited, and effective treatment strategies are relatively scarce. Therefore, the accurate and efficient detection of EGFR ex20ins mutations is crucial for treatment decisions. Next-generation sequencing (NGS) provides a more comprehensive approach to mutation detection compared to other methods like PCR (28, 35–37).

Currently, chemotherapy remains the standard first-line treatment (20, 45), with platinum-based chemotherapy, in particular, being the preferred choice (45). Immunotherapy has seen limited research in the context of this rare mutation, and its efficacy is not yet optimal, necessitating further exploration of its potential (49). Due to the structural peculiarities of EGFR ex20ins mutations (20, 21), their affinity for traditional EGFR TKIs is relatively low. Consequently, first-generation, and second-generation EGFR TKIs exhibit limited effectiveness against them. Although third-generation EGFR TKIs have shown some promising results, the associated data are relatively scarce. However, studies have suggested that the A763–764insFQEA mutation displays sensitivity to first, second, and third-generation EGFR TKIs (13, 16, 20, 100). The observed variations in sensitivity to EGFR TKIs may stem from differences in the location of the inserted sequence. Despite the suboptimal performance of conventional targeted therapies, recent advancements have introduced new targeted drugs. Earlier research results have indicated that patients treated with Mobocertinib (67, 68) and Amivantamab (15) achieved favorable objective response rates (ORR) and disease control rates (DCR), and these treatment methods are relatively safe. The U.S. Food and Drug Administration (FDA) has approved Mobocertinib and Amivantamab for the treatment of late-stage or metastatic non-small cell lung cancer patients with EGFR ex20ins mutations (28, 29). Although the results of the Phase III EXCLAIM-2 study fell short of expectations, leading to the withdrawal of Mobocertinib, other drugs, such as the domestically developed third-generation EGFR TKI Furmonertinib (80) and DZD9008, have shown outstanding anti-tumor activity in EGFR ex20ins patients, particularly those with brain metastases (69, 82, 84). DZD9008 has demonstrated the highest ORR and is poised to become a primary treatment choice, with the FDA granting it Breakthrough Therapy Designation (BTD). While Poziotinib exhibits some anti-tumor activity, its safety issues remain unresolved, and it has not yet seen widespread clinical use (74).

Moreover, not all Exon 20ins mutations respond similarly to TKIs. Studies have indicated that some mutations may confer resistance or reduced sensitivity to certain TKIs, while others may exhibit greater responsiveness, depending on the structural domains where the insertion mutations occur (30). It is evident that the differences in mutations at different sites highlight the subtleties in understanding treatment outcomes. In the CHRYSALIS trial, Amivantamab demonstrated a higher response rate to mutations closely following the α-C helix (codons 767–771) compared to those located in the distal loop (codons 771–775). In a trial evaluating Poziotinib, it was found that proximal loop insertions were more sensitive compared to distal loop insertions, with respective ORRs of 46% and 0% (p = 0.0015). Similar results have been observed with drugs like Mobocertinib, Zipalertinib, and JMT101. Both Furmonertinib and Sunvozertinib exhibited drug sensitivity irrespective of mutation sites. In the EXOTIC trial (43), 83% of mutations were located in the proximal loop, and 4% were within the helix, suggesting that novel targeted inhibitors are expected to have high activity in treating Exon 20 insertion mutations in nearly 90% of cases. Given the varying responses of different EGFRex20ins variants to EGFR TKIs, treatment strategies tailored to different EGFRex20ins variants may be needed to maximize TKI efficacy. In 2021, Heymach et al. proposed a structural-functional reclassification of EGFR mutations to enhance the prediction of drug efficacy (101). Zwierenga et al. suggested that the operability of individual EGFR ex20 mutations is heterogeneous, with TKI efficacy and sensitivity in Ba/F3 and patient-derived cell lines consistent with clinical data from *in vitro* studies (102). For instance, mutations at the commonly affected amino acid position A767 in Ba/F3 cells were sensitive to Poziotinib, Osimertinib, Zipalertinib, and Mobocertinib. Consistent with these findings, patient-derived cell lines and patients carrying these mutations were also sensitive to Osimertinib (160 mg) and Mobocertinib. Previous studies have shown that researchers create appropriate drugs for different mutations based on homology modeling and simulation experiments (103). In future work, focus should be placed on exploring structural changes to determine which mutation sites render patients more sensitive to EGFR TKIs, whether accompanied by alterations in other pathways, to provide a basis for prognosis assessment and drug decision-making (104).

With the continuous progress in new drug development, there is still significant research potential in this field. Ongoing clinical studies are providing new treatment options for non-small cell lung cancer patients with EGFR ex20ins mutations and paving the way for future therapeutic possibilities.

However, there is currently a paucity of clinical research and data regarding effective first-line treatment for late-stage untreated NSCLC patients with EGFR ex20ins mutations. Most studies have been conducted on patients who have progressed after treatment with platinum-based chemotherapy, immunotherapy, or EGFR TKIs. The sensitivity to drugs may vary depending on the specific insertion mutation location, warranting further research. There is also potential for further investigation into other drugs. Despite the advancements achieved with newly developed targeted therapies, resistance inevitably arises during the treatment course (105). Exploring resistance mechanisms, overcoming resistance, and improving treatment precision and safety are also areas of focus. Precise treatment targeting the specific mutation site can reduce adverse events. Due to the uncertainty and complexity of resistance development, significant investment in time, manpower, and resources is required. Currently, identified resistance mechanisms in clinical samples include PIK3CA E545K, MAP2K2 S94L, MET amplification, EGFR amplification, and CDK6 amplification. In drugs such as Poziotinib, Mobocertinib, Zipalertinib, and Osimertinib, the emergence of the C797S mutation may promote clinical resistance by covalently binding to the EGFR ATP-binding site (88). In this regard, the use of Hsp90 inhibitors alone or in combination with other therapies may yield unexpected therapeutic effects (106). For other drugs, resistance mechanisms remain unclear due to limited research. It is speculated that resistance mechanisms in some small molecule TKIs may arise from changes in kinase structural domains or activation of bypass signaling pathways. In the future, besides focusing on developing novel drugs suitable for different mutation sites, we should also analyze the mechanisms underlying disease progression or resistance caused by different drugs. Additionally, efforts can be directed toward understanding the reasons behind varying treatment outcomes and developing new drugs to address these causes or exploring combination therapy strategies with complementary mechanisms of action.

In summary, the future holds promise for more new drugs and treatment strategies, offering personalized, precise, and safe treatment for NSCLC patients with EGFR ex20ins mutations.

## Author contributions

XM: Writing – original draft, Writing – review & editing, Data curation, Methodology, Formal analysis. XS: Writing – original draft, Data curation. CC: Writing – original draft, Data curation. YX: Writing – original draft, Data curation. JZ: Writing – original draft, Data curation. LY: Writing – review & editing.
